# Magnetic, Electronic Structure and Micromagnetic Properties of Ferrimagnetic DyCo_3_ as a Platform for Ferrimagnetic Skyrmions

**DOI:** 10.3390/nano15080606

**Published:** 2025-04-15

**Authors:** Radu George Hategan, Andrei Aldea, Razvan Dan Miclea, Razvan Hirian, Ioan Botiz, Roxana Dudric, Lokesh Rasabathina, Olav Hellwig, Georgeta Salvan, Dietrich R. T. Zahn, Romulus Tetean, Coriolan Tiusan

**Affiliations:** 1 Faculty of Physics, Babeș-Bolyai University, 400084 Cluj-Napoca, Romania; radu.hategan@ubbcluj.ro (R.G.H.); andrei.aldea@stud.ubbcluj.ro (A.A.); razvan.miclea@ubbcluj.ro (R.D.M.); razvan.hirian@ubbcluj.ro (R.H.); ioan.botiz@ubbcluj.ro (I.B.); romulus.tetean@ubbcluj.ro (R.T.); 2 Institute of Physics, Chemnitz University of Technology, 09107 Chemnitz, Germany; lokesh.rasabathina@physik.tu-chemnitz.de (L.R.); olav.hellwig@physik.tu-chemnitz.de (O.H.); salvan@physik.tu-chemnitz.de (G.S.); zahn@physik.tu-chemnitz.de (D.R.T.Z.); 3 Center of Materials, Architectures and Integration of Nanomembranes, Chemnitz University of Technology, 09107 Chemnitz, Germany; 4 National Center of Scientific Research, 54000 Nancy, France

**Keywords:** rare earth–transition metal ferrimagnetic structures, skyrmions, skyrmionic materials, micromagnetism, spintronics, Dzyaloshinskii–Moriya asymmetric exchange

## Abstract

We demonstrate tunable ferrimagnetic properties in both bulk and thin film ferrimagnetic DyCo_3_ compatible with the hosting of topological magnetic chiral textures, namely skyrmions suitable for integration into spintronic applications with classic, neuromorphic and quantum functionalities. The bulk samples were prepared by arc-melting of stoichiometric mixtures under purified argon atmosphere and the thin films by Ultra-High-Vacuum magnetron sputtering from a stoichiometric target. Magnetometry allows us to extract the main magnetic properties of bulk and thin films: the saturation magnetization, the magnetic anisotropy and their variation with temperature. These results are successfully complemented by band structure ab initio DFT calculations. Based on the critical magnetic parameters extracted from experiments, we performed micromagnetic simulations that reveal the skyrmionic potential of our samples in both continuous thin film and nano-patterned architectures.

## 1. Introduction

Spintronics, which harnesses both the spin and charge properties of electrons [1], has transformed information technology and offers a promising solution for developing energy-efficient electronics that can overcome the limitations of Moore’s Law [2]. Traditional spintronics has relied on two key processes in ferromagnetic materials: information storage through magnetization control (‘writing’), and information retrieval through magneto-resistance effects (‘reading’). The field has since expanded to include ferrimagnetic and antiferromagnetic materials, the last class offering unique advantages due to their zero net magnetic moment. These materials’ absence of stray magnetic fields enables both higher storage density and faster operation—reaching terahertz (THz) frequencies—in information-processing devices [3]. The demand for increased storage density has driven spintronics toward a new generation of devices based on nanoscale chiral magnetic structures, particularly domain walls and skyrmions. Skyrmions—a distinct class of topologically protected solitons—can encode information in multiple ways: through their core polarization and chirality (for classical bits) or helicity (for quantum bits/qubits). These structures can be manipulated with minimal energy consumption, making them promising for a wide range of applications, including next-generation racetrack memories [4], neuromorphic computing [5], and quantum technologies [6,7,8].

For applications based on skyrmions, one needs to fabricate “skyrmionic materials” with enhanced properties in which they can be stabilized within a well-defined window in a phase-diagram space of magnetic properties [9] by the Dzyaloshinskii–Moriya interaction (DMI) competing with other energies: e.g., anisotropy, magnetostatic, direct exchange, and thermal fluctuations. Antiferromagnetic (AF) skyrmions are particularly promising for various applications. In classic devices, they eliminate the skyrmion Hall effect (SkHE) [10] during current-driven motion, as demonstrated in synthetic antiferromagnet devices [11]. For quantum computing, antiferromagnetic interactions in ferrimagnetic skyrmion materials can enable qubit coupling—crucial for scalability—through either interlayer exchange in multilayered structures [6] or intrinsic AF skyrmions in ferrimagnets and antiferromagnets [12]. In neuromorphic applications, Spin Transfer Torque (STT)-driven skyrmion nano-oscillators could operate at terahertz frequencies. Ferrimagnetic materials offer a compelling pathway forward by combining the advantages of both ferro- and antiferromagnets [13]. Their finite spin polarization enables spin current control, while the inequivalent antiferromagnetically coupled spin lattices allow fine-tuning of both saturation magnetization (M_s_) and angular momentum density (A). Unlike ferromagnets, where the gyromagnetic ratio (γ = M_s_/A) remains nearly constant with temperature, ferrimagnets offer independent control of M_s_ and A through temperature and composition adjustments. This creates distinct compensation points for angular momentum and magnetization, enabling unique control over magnetic properties. A particularly interesting situation occurs at the angular momentum compensation temperature where the magnetization is not compensated, and the ferro- and antiferromagnetic features are combined: antiferromagnetic magnetization dynamics is realized, but Zeeman coupling is finite. In this respect, ferrimagnets can serve as a material platform not only to investigate antiferromagnetic spin textures and dynamics but also to fabricate practical spintronic devices that exploit the advantages of both antiferromagnets and ferromagnets. Rare-Earth-Transition-Metal (RE-TM) ferrimagnetic alloys are particularly promising due to their tunable magnetic properties [14]. When grown as thin films, they provide the perpendicular magnetic anisotropy needed for high-density spintronic devices, while the RE’s strong spin–orbit coupling promotes substantial DMI. Recent studies have demonstrated skyrmion stabilization in these materials [12,15], showing reduced [15] or eliminated SkHE at specific temperatures [16]. Several DMI mechanisms have been identified in ferrimagnetic RE-TM alloy films with perpendicular magnetic anisotropy suitable for skyrmion hosting [17,18,19] of the bulk DMI from asymmetric elemental distribution [20], the weak interfacial DMI induced by proximity effects with adjacent layers [21] or the absence of the inversion symmetry center in amorphous materials, inherently leading to a non-vanishing DMI [22]. When interfaced with heavy metals (e.g., Pt), the interfacial DMI promotes the Néel-type spin textures, e.g., room-temperature skyrmions with diameters ≈100 nm in [Pt/SmCo_5_/Ta]_15_ multilayers or skyrmionium-like spin textures in [Pt/SmCo_5_/Ir]_15_ multilayers [23]. A first experimental observation of skyrmions in Nd_2_Fe_14_B has been unambiguously revealed by observation of Lorentz transmission electron microscopy at variable temperatures and magnetic fields, and explained by spin reorientation transition mechanisms [24]. For the case of DyCo_x_ alloys, few existing studies indicate the existence of intrinsic noncollinear magnetic ground states for both single-crystal and amorphous films [25,26,27]. One of the first experimental evidence of skyrmions in DyCo_3_, based on X-ray magnetic scattering, scanning transmission X-ray microscopy and Hall transport technique, revealed compact ferrimagnetic skyrmions with antiparallel Dy and Co moments, approximately 40 nm in radius, forming during magnetic domain pattern changes and exhibiting a topological Hall effect [12].

In our study, we use a multi-scale approach, combining theoretical modelling and experiments performed on DyCo_3_ RE-TM systems at two-dimensional scales in bulk and thin films. This approach addresses some complex issues related to DyCo_3_’s magnetic and electronic structure properties, having in view its already demonstrated potential for compact ferrimagnetic skyrmions stabilization. These two types of samples are prepared using the following methods: the bulk specimens via arc-melting of stoichiometric mixtures in purified argon atmosphere, and the thin films through ultra-high-vacuum magnetron sputtering from a stoichiometric target. Through magnetometry measurements, we determined key magnetic properties including saturation magnetization and magnetic anisotropy, as well as their temperature dependence. The experimental findings are corroborated by ab-initio density functional theory (DFT) calculations of the band structure. In thin films, we used magnetometry and Magnetic Force Microscopy to demonstrate their perpendicular magnetic anisotropy, which promotes remanent perpendicular maze-domain patterns. Combined with the DMI, this is one of the key requirements for skyrmion hosting in these materials. Micromagnetic simulations, based on experimentally determined magnetic parameters, reveal the magnetic parameter window allowing the formation of skyrmions in both continuous films and nanopatterned structures, an important issue for both classical, neuromorphic and quantum applications. In the absence of direct DMI evaluation, with Brillouin Light Scattering experiments being a future prospect, we treat the DMI magnitude as a tunable parameter in our models. This can be experimentally adjusted by modifying the material’s structural, morphological, and crystallographic properties. Given the DMI’s critical role in skyrmion stabilization, our micromagnetic simulations and phase diagrams identify the DMI range required for skyrmion formation when the PMA criteria are met. The precise control of the materials properties window (namely for the PMA and DMI) in which the ferrimagnetic skyrmions can be stabilized and manipulated represents a major need for potential classic and quantum applications. Recent studies increasingly highlight the significance of antiferromagnetic and ferrimagnetic skyrmions in skyrmionic applications, such as racetrack memories, where they enable the vanishing of the Skyrmion Hall Effect and enhance the spin-current-induced velocity [28,29,30]. These skyrmions also improve the performance of spin-torque nano-oscillators, operating at frequencies of several tens of GHz, an order of magnitude higher than their ferromagnetic counterparts [31,32].

## 2. Materials and Methods

The DyCo_3_ samples have been prepared in bulk and thin film configurations, using two different methods. The bulk samples were obtained by arc-melting stoichiometric proportions of high purity Dy and Co (Sigma-Aldrich, St. Louis, MO, USA) in an argon atmosphere. The sample was turned and melted again multiple times to achieve a homogenous composition. The ingot was then wrapped in a tantalum foil and annealed in a quartz tube under high vacuum (10^−^^5^ mbar) at 900 °C for 5 days. The thin films were prepared by Ultra-High-Vacuum (UHV) sputtering from a 2 inch (5.08 cm) stoichiometric DyCo_3_ target with Dy:Co 1:3 atomic ratio and 99.9% purity. The base pressure in the sputtering chamber, prior to the deposition was in the range of 10^−^^9^ Torr, and the Ar pressure during the plasma deposition was approximately 3.3 mTorr. The thin films were deposited using DC magnetron sources with an optimized power of 75 W providing the lowest film roughness. The deposition rate, measured with a quartz balance during the deposition, was in the range of 0.8–1 nm/s. The DyCo_3_ films were sputtered on thermally oxidized Si(100) substrates and protected by a Ta capping.

The structural characterization of the bulk polycrystalline samples was performed at room temperature via X-ray powder diffraction on a Bruker D8 Advance diffractometer equipped with a CuKα source (Bruker AXS, Karlsruhe, Germany). The structural parameters were obtained through the Rietveld refinement of the experimental diffraction pattern via FullProf software (Laboratoire Léon Brillouin CEA-CNRS, Centre d’Etudes de Saclay, Gif sur Yvette, France, Institut Laue-Langevin, Grenoble, France). Given the thickness of the thin films (around 100 nm), the goniometric geometry available in our diffraction experiments did not allow a signal-to-noise ratio suitable for structural analysis. However, the topological quality of the thin films was extensively analyzed using Atomic Force Microscopy (AFM). The AFM measurements were conducted with an AFM Microscope from Molecular Devices and Tools for Nano Technology (NT-MDT), which was mounted on an Olympus IX71 optical microscope (Spectrum Instruments Ltd., Limerick, Ireland) and operated in tapping mode. High-resolution Noncontact Golden Silicon probes from NT-MDT were used, with a scanning speed of approximately 1–2 μm/s. The set point was adjusted between 9–12 V for each sample to ensure a soft tapping regime. Magnetic Force Microscopy (MFM) imaging was performed using a magnetic force Bruker Dimension Icon microscope using MESP-Brucker Co-Cr coated tips. In tapping mode, the trace line reveals the surface topography through short-range van der Waals interactions, while the retrace line scan, conducted at a 15 nm lift distance above the surface, provides magnetic information through longer-range magnetic interactions with the tip and domain structures.

The magnetic characterization of the bulk samples has been performed on a Cryogenic Ltd. (London, UK) Vibrating Sample Magnetometer (VSM) operating in a temperature window of 4 to 300 K and external applied magnetic field of up to 12 T. The spontaneous magnetization *M* was determined by fitting the experimental magnetization curves with an approach to saturation type law [33]: $M=M_{s}\left(1-a/B-b/B^{2}\right)+\chi B$, where *B* is the magnetic field, M_s_ the saturation magnetization, *a* the coefficient of magnetic hardness, *b* a constant related to the magnetic anisotropy, and χ is a Pauli-type contribution. The effective anisotropy of the samples has been extracted from the measured saturation field, using the equation $K_{u}=K_{eff}=\frac{B_{s}M_{s}}{2}$ issued from the standard Stoner–Wohlfarth model. On the other hand, the magnetic properties of the thin films were determined using a Quantum Design MPMS (Magnetic Property Measurement System) SQUID-VSM, providing a higher sensitivity to magnetic moment, a maximum magnetic field of 7 T and a temperature range of 1.8 K–1000 K. Beyond the models employed for bulk material magnetic analysis, for the thin films we additionally employed the model of Thiele [34], allowing us to quantify micromagnetic features corresponding to out-of-plane anisotropy experimentally found in the thickness range of our sample. This model allows us to correlate the in-plane and out-of-plane saturation fields of the film with characteristic magnetic lengths, material magnetic properties (nominal thickness, anisotropy, saturation magnetization) and domain wall features. Roughly, within the model of Thiele, the saturation field when the field is applied perpendicular to the sample hosting stripe domains due to the perpendicular anisotropy is given by the following equation:

(1) $$H_{s}^{⊥}=4\pi M_{s}\left(1-1.596\sqrt{\frac{l}{h}}\right)$$

where $M_{s}$ is the saturation magnetization, $l=\frac{\sigma_{w}}{4\pi M_{s}^{2}}$ represents a characteristic length and $\sigma_{w}=4\sqrt{AK}$ the domain wall width.

The saturation field when the field is applied within the plane of the film is given by the following:

(2) $$H_{s}^{∥}=\frac{2}{M_{s}}\left(K_{1}-2\pi N_{d}M_{s}^{2}\right)$$

with $N_{d}$ being a demagnetizing factor corresponding to a given ratio between the width of the magnetic stripe and the thickness of the film w/h [35]. These equations have been used to estimate the perpendicular anisotropy from the in-plane and out-of-plane magnetization loops.

The band structure calculations were performed using the Full Potential Linearized Augmented Plane-Wave method (FPLAPW)+local orbitals (lo) implemented in the Wien2k ab initio code [36] within a fully relativistic scheme. The Local Spin Density Approach (LSDA) + U Hubbard has been considered. The effective value of U has been chosen as **U_eff_** = **U** − **J** = **0.5 Ry** after performing a complete convergence study for the energy, orbital and spin moment on Dy and Gd as a function of U and J. The chosen convergence criterion was the maximization of the Dy orbital momentum. The size of the basis set used in the wave function set expansion was $R_{MT}\cdot K_{max}=7$, where R_MT_ is the smallest atomic muffin-tin sphere radius and K_max_ the largest K-vector, corresponding to a cutoff energy of 7.84 Ry. A fully relativistic scheme has been considered, including the spin–orbit interaction term in the Hamiltonian. Prior to any band structure analysis, we have performed a thorough optimization of the structural parameters, relaxing the atomic positions to minimize the total energy. The calculation of the magnetocrystalline anisotropy energy (MAE) was carried out using a fully relativistic spin–orbit scheme, applying both the total energy and force theorem approaches [37], which yielded similar results. In the total energy approach, which incorporates spin–orbit interactions, the magnetic anisotropy energy was estimated as the total energy difference between the easy direction (magnetization perpendicular to the film plane) and the hard direction (in-plane magnetization). Given the high sensitivity of the magnetic anisotropy energy to the k-space meshing, a thorough convergence study of the total energy with respect to the number of k-points was first conducted, leading to an integration scheme in k-space based on a modified tetrahedron method with 1000 k-points within an irreducible Brillouin zone.

Micromagnetic simulations were performed using the GPU-accelerated Mumax3 code (Ghent University) [38], which solves the Landau–Lifshitz–Gilbert (LLG) equation:

$$\frac{dm}{dt}=-\frac{\gamma }{1+\alpha^{2}}\left(m\times B_{eff}\right)-\frac{\alpha \gamma }{1+\alpha^{2}}\left[m\times \left(m\times B_{eff}\right)\right]$$

where **B_eff_** is the effective field, α is the Gilbert damping parameter, γ is the gyromagnetic ratio (1.75 × 10^7^ s Oe-1) and μ_0_ the vacuum permeability (4π × 10^−^^7^ F/m). The effective field **B_eff_** is computed as the functional derivative of the magnetic free energy of the system *E[m,t]* that includes contributions from Zeeman, demagnetizing, anisotropy, exchange (symmetric *J_0_* and asymmetric Dzyaloshinskii–Moriya interaction), etc.: $B_{eff}=-\frac{1}{M_{s}}\frac{ðE}{ðm}$. The simulations were conducted on an NVIDIA Geforce RTX 3070 graphics card with 5888 CUDA cores for parallel computing. The modeled systems consisted of either continuous film structures or circular nano-disks with a given diameter and thickness. The magnetic anisotropy was introduced as a 1st order uniaxial anisotropy K_u_ along the [001] axis, and the DMI has been chosen to have bulk origin (**D_bulk_**), having in view the thickness of the simulated films, above 100 nm. Material and multilayer stack parameters, including the Gilbert damping parameter α, exchange stiffness, **A_ex_** and saturation magnetization M_s_ were also incorporated. For dynamical simulations, the LLG equation was integrated using the adaptive-step Runge–Kutta method (RK45) with the Dormand–Prince solver. In the calculation of phase diagrams, the energy minimum of the states was obtained using the Mumax3 function *relax()*, which disables the precession term of the LLG equation and allows the system to relax toward its energy minimum. To account for temperature effects, a finite temperature was applied to the ground state after relaxation. The evolution and stability of the system were then studied over a finite time integration window. The classification of the final micromagnetic states was performed by calculating the topological charge: $Q=\frac{1}{4\pi }\int dxdy\vec{m}\cdot \left(\frac{d\vec{m}}{dx}\times \frac{d\vec{m}}{dy}\right)$.

## 3. Results

### 3.1. Ab Initio Calculations of DyCo_3_

**Figure 1:**
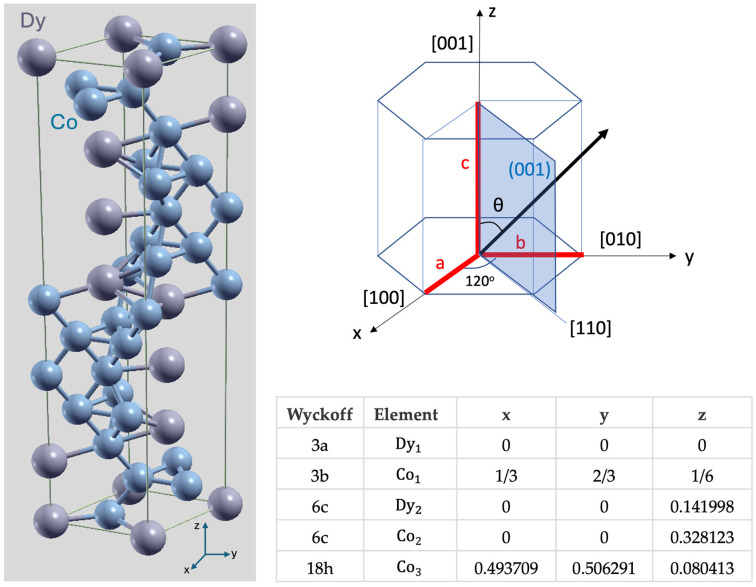
Supercell model used in the ab initio calculations corresponding to a hexagonal symmetry cell. The table depicts the crystallographic positions of the atoms corresponding to the Wyckoff notations.

The ab initio calculations allowed us to obtain a deeper understanding of the main mechanisms responsible for the magnetic properties of the DyCo_3_ compound, correlated to its structural characteristics. Our calculation is based on a supercell model, as illustrated in Figure 1, based on the specific crystallographic characteristics of DyCo_3_. This compound is Frank–Kasper μ phase-like structured, and crystallizes in the trigonal R$\bar{3}$m space group. The Dy occupies two inequivalent sites. In the first Dy site, Dy is bonded in a 12-coordinate geometry to twelve Co atoms. There is a spread of Dy–Co bond distances ranging from 2.91–3.06 Å. In the second Dy site, Dy is bonded in an 18-coordinate geometry to eighteen Co atoms. There are six shorter (2.90 Å) and twelve longer (3.16 Å) Dy–Co bond lengths. There are three inequivalent Co sites. In the first Co site, Co is bonded to five Dy and seven Co atoms to form CoDy_5_Co_7_ cuboctahedra that share corners with seventeen CoDy_6_Co_6_ cuboctahedra, edges with eight equivalent CoDy_5_Co_7_ cuboctahedra, and faces with fourteen CoDy_5_Co_7_ cuboctahedra. There is a spread of Co–Co bond distances ranging from 2.35–2.60 Å. In the second Co site, Co is bonded in a 9-coordinate geometry to three equivalent Dy and six equivalent Co atoms. In the third Co site, Co is bonded to six equivalent Dy and six equivalent Co atoms to form CoDy_6_Co_6_ cuboctahedra that share corners with twelve equivalent CoDy_5_Co_7_ cuboctahedra, edges with six equivalent CoDy_6_Co_6_ cuboctahedra, and faces with eighteen equivalent CoDy_5_Co_7_ cuboctahedra.

**Figure 2:**
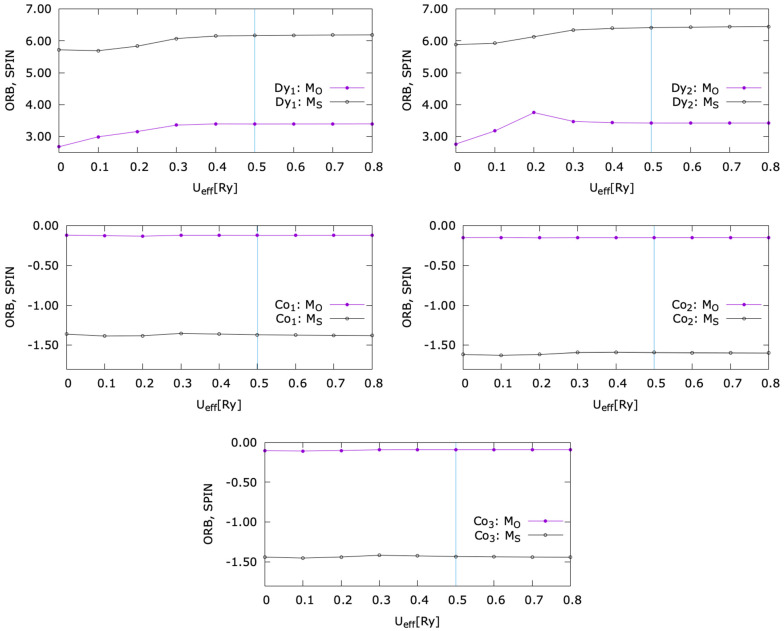
Orbital and spin magnetic moment variation with the Hubbard parameter U for each inequivalent Dy and Co atom in the supercell slab used for the ab initio calculation. The choice for further ab initio analysis is U_eff_ = 0.5 Ry, indicated on each figure, at which the Dy orbital moment is saturated.

As mentioned in the Methods section, the first steps in the ab initio calculations were as follows:

(i) finding the theoretical atomic equilibrium position by performing a structure relaxation to minimize total energy and force;
(ii) a careful convergence in k point meshing;
(iii) a study concerning a choice of the Hubbard U parameter further used for calculations. The choice criterium for the U was a value larger than the one that maximizes the orbital momentum of Dy. As illustrated by Figure 2, the adjunction of the Hubbard correlations leads to an increase of the Dy orbital moment which eventually saturates after a certain value of U. As expected, the Hubbard correlation influence is negligible for Co: the magnetic moment of the Co atoms is mostly independent on U. Moreover, as expected for a transition metal, the orbital contribution to the total magnetic moment is negligible as compared to the spin one.

**Figure 3:**
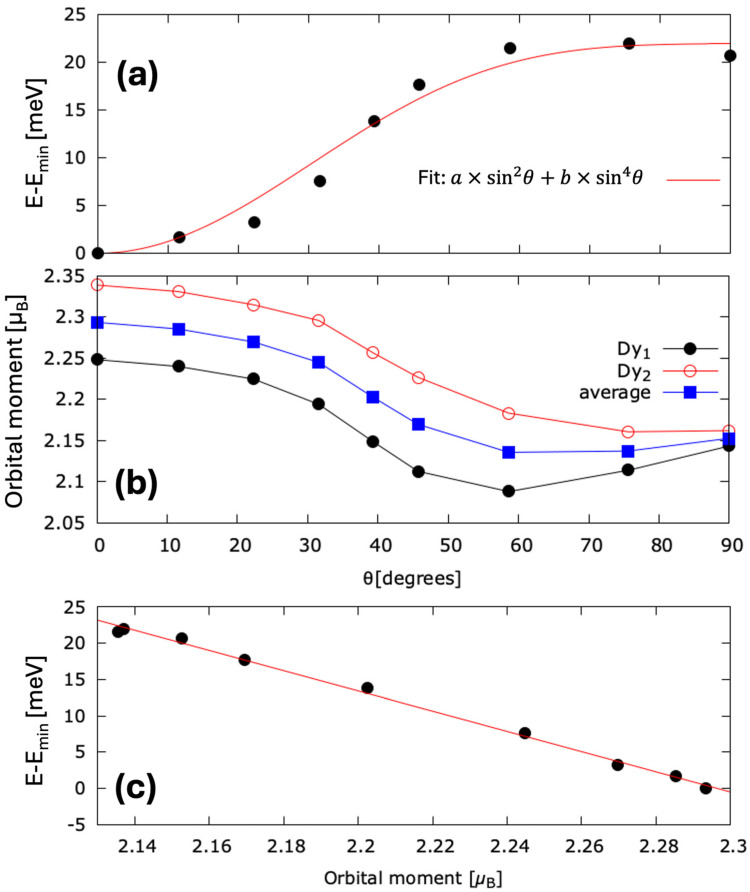
(**a**) Magnetic anisotropy energy calculated as total energy difference between two orientations of the magnetization corresponding to θ=0 and θ with respect to the [001] hexagonal c axis. (**b**) Orbital momentum anisotropy for each of the two inequivalent Dy atoms, and for the average value. (**c**) Relative energy dependence with respect to the orbital momentum, illustrating that the minimum of energy corresponds to the maximum of L.

Considering the fully relativistic spin–orbit contribution to the Hamiltonian implemented in *Wien2k*, we were able to tackle the anisotropy analysis. The anisotropy energy calculated as the difference between total energy corresponding to different orientation θ of the magnetization with respect to the crystallographic z axis (see Figure 1) is estimated in a perturbation scheme and represented in Figure 3a.

Our results confirm theoretically that DyCo_3_ has a hexagonal anisotropy according to its hexagonal structure, with an easy axis parallel to the [001] c hexagonal axis (see Figure 1). Moreover, our calculations clearly underline the correlation between the monocrystalline anisotropy and the orbital momentum anisotropy (Figure 3b,c): the system has an energy minimum when the magnetization has an orientation that maximizes the orbital momentum. This result is in very good agreement with the theoretically expected orbital momentum anisotropy, as predicted by Bruno [39].

In Table 1, we summarize the spin and orbital magnetic moment for the inequivalent Dy and Co atoms, the calculation being performed with an effective Hubbard term $U_{eff}=U-J=0.5$ Ry.

From these magnetic data, we can estimate the total theoretical magnetic moment per formula unit, considering the Wyckoff occupation of each site: M[f.u.] = (3 × 9.61 + 6 × 9.98 − 3 × 1.49 − 6 × 1.74 − 18 × 1.52)/9 = 5.16 μ_B_/f.u.

### 3.2. Comparison with the Experiments

The Wien2k framework, based on density functional theory (DFT), provides a good estimation for magnetic moments, but it may not capture the full complexity of the experimental magnetic configurations. This is especially true for complex systems like DyCo_3_, where the magnetic interactions are influenced by multiple factors, such as spin–orbit coupling, exchange interactions, and anisotropy, which might not be fully captured by a limited Hamiltonian model. From the experimental data provided by neutron diffraction [40], we know that the moments of Dy are aligned at 36.9° and Co at 211° with respect to the c-axis. This indicates a non-collinear, ferrimagnetic arrangement, which suggests complex interactions between the two magnetic sublattices (Dy and Co). Even though the theoretical model does not perfectly match the experimental configuration, one can still consider the theoretical results as a reasonable expectation, within the limits of the model. The predicted magnetic moment per formula unit could serve as a useful starting point or approximation, providing insights into the general trends of the system, but it should be taken with some caution for a full comparison to experimentally determined values. Moreover, the theoretical model sheds light on the intrinsic mechanisms responsible for some major magnetic issues: the ferrimagnetic ground state of the DyCo_3_ system is determined by the coupling between the Dy and Co mediated by the hybridization between the 5d electrons of Dy and 3d electrons of the Co, the magnetic moments of Dy and Co are strongly dependent on their local coordination geometry and bonding and have inequivalent values corresponding to the inequivalent site. These properties allow us to intuitively understand the impact of the local atomic environment on the magnetic moment and, consequently, the relatively large dispersion on the values reported in the literature for the saturation magnetic moment per formula unit for DyCo_3_, from 4.3–7.78 μ_B_/f.u.) [41,42,43,44].

### 3.3. Structural Properties of the Bulk DyCo_3_

The X-ray diffraction pattern of the annealed sample revealed that the sample crystallized in the expected rhombohedral PuNi_3_-type structure, R$\bar{3}$m space group.

**Figure 4:**
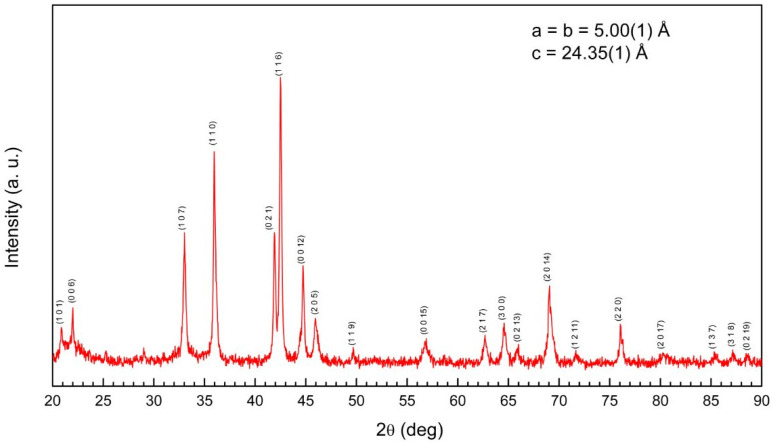
Indexed diffraction diagram (θ–2θ scan) corresponding to the bulk DyCo_3_ samples.

The experimental diffraction pattern and the position of reflections corresponding to different crystallographic planes indexed in the Rietveld refinement are shown in Figure 4. The lattice parameters (see Figure 4), determined from the refinement of the experimental data, are in very good agreement with previously reported values [44,45]. Within the limit of the experimental errors, this would indicate that our bulk DyCo_3_ compound is stoichiometric, does not contain any additional phases and has the expected crystallographic structure [38,39].

### 3.4. Magnetic Properties of the Bulk DyCo_3_ Systems

**Figure 5:**
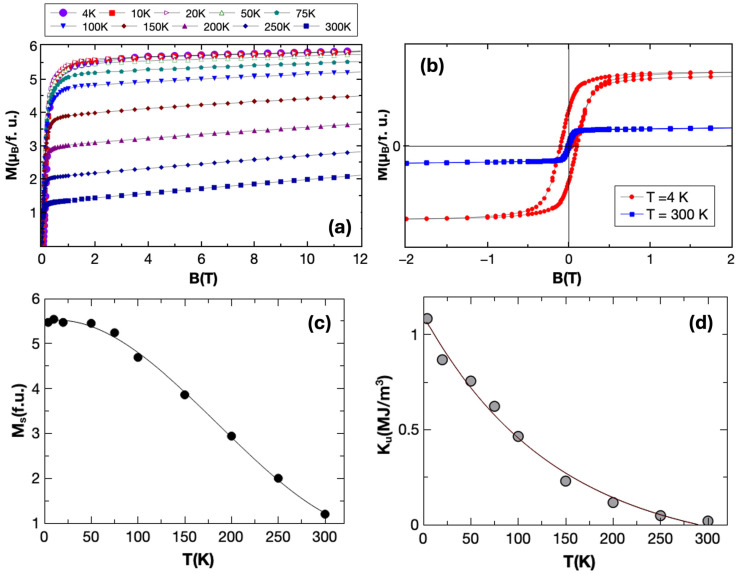
(**a**) Magnetization isotherms measured at different temperatures within the range 4 K–300 K. (**b**) Typical M-B loops, at 4 and 300 K, respectively. (**c**) Magnetization [f.u.] dependence on temperature. (**d**) Effective uniaxial anisotropy dependence on temperature, calculated from the saturation field and using a Stoner–Wohlfarth approach.

The vibrating sample magnetometry experiments performed on the bulk samples are illustrated in Figure 5.

The magnetization isotherms measured in a field range of 0–12 T and chosen temperatures within the range 4–300 K are depicted in Figure 5a, while Figure 5b illustrates typical M-B loops at extreme temperatures of 4 K and 300 K. As described in the Methods section, these experiments allow the extraction of the saturation magnetization M_s_ in Bohr magnetons per formula unit by fitting the data to the law of approach to saturation, as shown in Figure 5c. Additionally, the saturation field *B*_s_ can be determined. Then, based on the Stoner–Wohlfarth model, the effective uniaxial anisotropy K_u_ can also be extracted, as illustrated in Figure 5d. From this type of analysis, we get access to important magnetic parameters of the material such as M_s_ and K_u_ and their variation with temperature, useful for further micromagnetic simulations. The saturation magnetization at 4 K is estimated to be around 5.4 $\mu_{B}$/f.u., a value that is in good agreement with the theoretical estimation (see Section 3.1 [41,42,43,45]). Moreover, this value can be explained, considering an antiparallel alignment of Dy with an experimental average moment of 10 $\mu_{B}$, according to the literature [44], with a Co magnetic moment having an average value of 1.53 $\mu_{B}$, a fact that confirms that our compound is in a ferrimagnetic state. These values are in good agreement with our theoretical estimations $\langle M_{Dy}\rangle =9.85\;\mu_{B}$ $\langle M_{Dy}\rangle =-1.57\;\mu_{B}$.

### 3.5. DyCo_x_ Thin Film Systems

**Figure 6:**
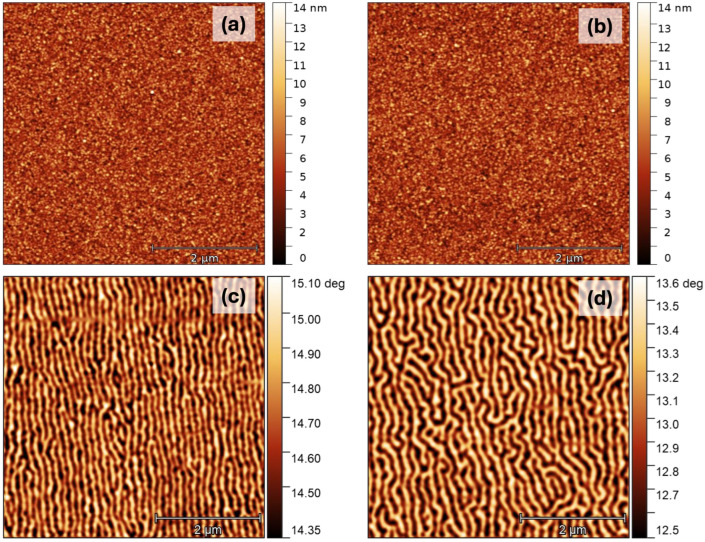
(**a**,**b**) Atomic force microscopy image corresponding to a 120 nm thick DyCo_3_ film capped with 12 nm of Ta. (**c**,**d**) Magnetic Force Microscopy images corresponding to in-plane and out-of-plane sample demagnetization with 96% as demagnetization factor. The MFM scan is performed at a height of 15 nm above the surface.

To advance applications beyond bulk systems, we fabricated and investigated the magnetic properties of DyCo_x_ thin films using an Ultra-High-Vacuum sputtering system with a base pressure in the range of 10^−^^9^ Torr. The films are grown by DC sputtering in Ar under a pressure of 3.3 mTorr from stoichiometric DyCo_3_ targets on thermally oxidized Si substrates and protected with a Ta capping layer. An optimum DC power of 75 W was found to provide the lowest film roughness, corresponding to an RMS value of 0.95 nm, as determined from AFM experiments illustrated in Figure 6a,b. The AFM image corresponds to a nominal DyCo_x_ film thickness estimated from magnetometry and X-ray Reflectivity experiments to be about 120 nm; that was our thickness choice for the magnetic studies and micromagnetic simulations. In parallel, in Figure 6c,d we illustrate the magnetic force microscopy images corresponding to the topographic images. The magnetic contrast confirms that in the demagnetized state (in-plane and out-of-plane demagnetization) the 120 nm thick DyCo_x_ sample is clearly hosting perpendicular magnetized domains, as expected for a thin film with perpendicular magnetic anisotropy (PMA), a fact further confirmed by the magnetization loops experiments. The maze-like domain wall structure induced by the PMA is a key factor in the nucleation of magnetic bubbles when a perpendicular magnetic field is applied. Furthermore, in the presence of DMI in the samples, these bubbles will take the form of skyrmions, with their chirality determined by the sign of the DMI. In our study, we had no access to the experimental evaluation of the DMI in our samples; Brillouin Light Scattering experiments were one of the prospective scheduled studies which were not carried out. Furthermore, applying a perpendicular magnetic field to promote skyrmions from maze domains during MFM experiments would be technically challenging due to the large field values required and their direct impact on the MFM tip. Moreover, MFM characterization would not provide a clear distinction between possible topological textures such as bubbles, skyrmions, and antiskyrmions, with Lorentz microscopy being a potential future approach in our study [46]. For these reasons, as outlined in the final section of the paper, we used micromagnetic simulations to predict the skyrmion potential of our films, based on the magnetic properties we were able to evaluate. In the absence of direct experimental data, we assumed a typical DMI value for continuous films under an applied perpendicular magnetic field and used theoretical phase diagrams to illustrate the conditions needed to achieve a skyrmionic ground state in patterned nanopillars without a magnetic field.

**Figure 7:**
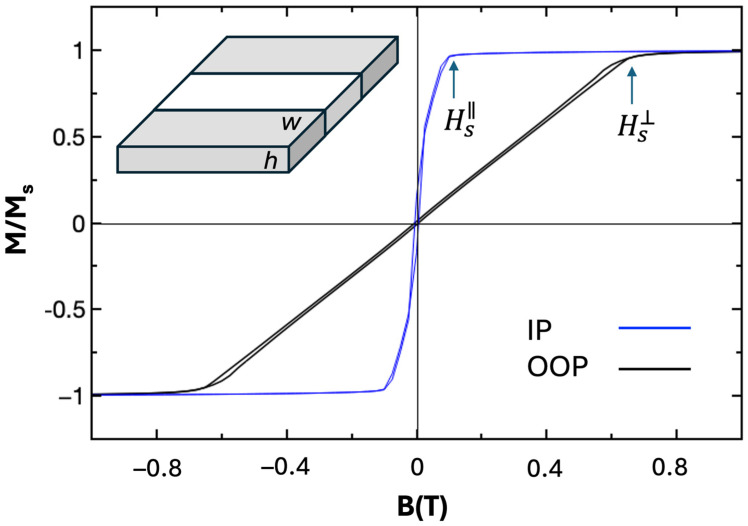
Room temperature magnetization curves M-B corresponding to the configurations in which the magnetic field has either in-plane (IP) or out-of-plane (OOP) orientation with respect to the magnetic film. Insert: stripe domain geometry leading to a demagnetizing factor N_d_ corresponding to a given w/h ratio [35].

Some typical magnetization *M-B* loops obtained by SQUID magnetometry are illustrated in Figure 7, corresponding to a measurement temperature T = 300 K.

We focus on the properties of the film at room temperature, as skyrmions are desirable for applications at this temperature. The magnetization curves at 300 K lead to an estimation of the saturation fields: $H_{s}^{⊥}\approx$ 0.65 T and $H_{s}^{∥}\approx$ 0.11 T and a saturation magnetization at room temperature of $M_{s}$ = 832 kA/m. Based on these parameters, using the framework of the Thiele model, as briefly mentioned in the Methods section, we can estimate the perpendicular anisotropy K_u_ ≈ 0.35 MJ/m^3^ and a demagnetizing factor N_d_ ≈ 0.7 corresponding to a *w*/*h* ≈ 0.3 ratio, following the model of Chen et al. [35].

These findings are particularly important for the micromagnetic simulations that we further performed to demonstrate that the experimental properties of our DyCo_x_ films are compatible with skyrmion hosting, an important pre-requisite for skyrmionic applications. Concerning the saturation magnetization, M_s_, the estimated value at 4 K from M-B analysis was around 956 kA/m, the SI unit choice in kA/m being for compatibility reasons with respect to the micromagnetic *Mumax3* code [38] used for simulations. The M_s_ = 956 kA/m value at 4 K corresponds to 6.03 **μ**_B_/f.u., larger than the one that we determined for the bulk DyCo_3_ samples (5.40 **μ**_B_/f.u.).

A possible explanation for this larger value of M_s_ observed in our sputtered thin films could be reasonably related to a sub-stoichiometric content of Co with respect to the real stoichiometry of the target (Dy:Co = 1:3). This could be induced by the sputtering yield of Dy that is larger than that of Co, mainly because the Dy binding energy is smaller than that of Co. Hence, aside from the anticipated increase in perpendicular anisotropy with film thickness in films exhibiting hexagonal symmetry [47] and considering that the Perpendicular Magnetic Anisotropy (PMA) combined with DMI is crucial for skyrmion formation, we selected relatively thick DyCo_x_ films (120 nm) with the expectation of a stoichiometry adjustment during the deposition process. At the beginning, the species with higher sputtering yields are sputtered more efficiently, which causes a depletion of those elements on the target surface. This depletion would, in turn, help compensate for the lower sputtering yield of other species, gradually correcting the stoichiometry. However, despite this expectation, a simple calculation based on our theoretically calculated average moments for Co and Dy—$\langle M_{Dy}\rangle =9.85\;\mu_{B}$ and $\langle M_{Co}\rangle =-1.57\;\mu_{B}$—results in a DyCo_x_ film with an effective x = 2.43. Therefore, for accuracy, we can consider that the magnetic properties experimentally determined from magnetometry would rather correspond to a DyCo_2.43_ film. Moreover, having in view the significant saturation magnetization of our DyCo_x_ films at room temperature (6.03 **μ**_B_/f.u.), we can reasonably argue that they are mono-phasic but sub-stoichiometric in Co. A mixture of DyCo_3_ and DyCo_2_ is excluded, due to the Curie temperature of the DyCo_2_ being well below room temperature RT (around 169 K) [45], which would imply a significant reduction of M_s_ at RT.

### 3.6. Micromagnetic Properties of DyCo_x_ Thin Films and Patterned Structures

Drawing from the experimental magnetic properties of both the bulk and thin film systems, we conducted micromagnetic simulations to show that these materials are well-suited for hosting skyrmions, making them promising candidates for skyrmion-based applications in information technologies.

**Figure 8:**
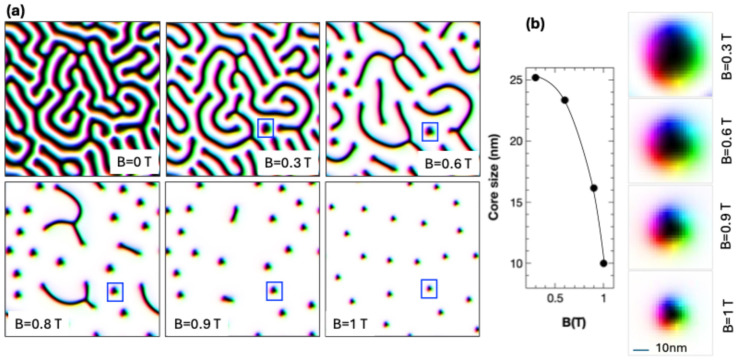
(**a**) Micromagnetic simulation of domain structure in continuous 120 nm thick DyCox films with the experimental magnetic parameters extracted from SQUID magnetometry. At zero magnetic field, the structure is perpendicular maze-type magnetized. With increasing field, skyrmions nucleate and form a non-regular network. (**b**) The size of the skyrmions is controlled by the field magnitude. The skyrmion subjected to the field-dependent study is the one indicated in figure (**a**) by a rectangle.

In Figure 8, we illustrate the results of a micromagnetic simulation corresponding to a continuous DyCo_x_ film with a chosen thickness of 120 nm, that mimics the experimental situation. The calculation was done using in-plane periodic boundary conditions N_x_ = N_y_ = 10, N_z_ = 1 and a parallelepipedal elementary cell of 512 × 512 × 124 nm with a cubic meshing unit cell of 2 nm lateral size. The simulations illustrate that the ground state of the film is a maze-type domain wall structure with perpendicular up (white) and down (black) orientation of magnetization (Figure 8). The skyrmions are progressively nucleated from these maze domains when a perpendicular magnetic field is applied. Moreover, the simulations clearly illustrate that the density and the size of the skyrmions can be controlled by the strength of the applied perpendicular magnetic field.

**Figure 9:**
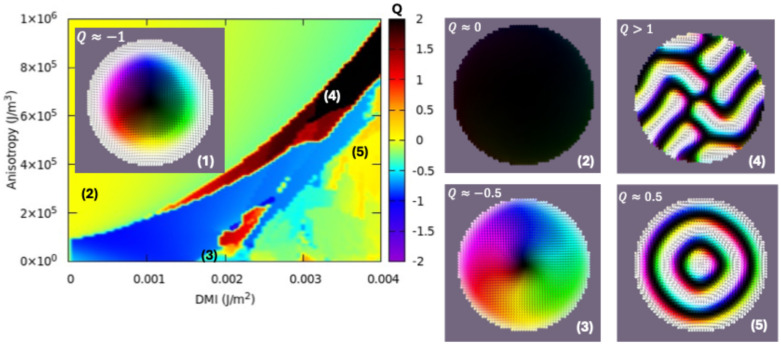
(**left**) Ground state DMI-Anisotropy-Topological charge calculated for a 100 nm diameter DyCo_x_ disk with thickness of 120 nm in which the micromagnetic states are classified by their topological charge Q. (**right**) Micromagnetic configuration in vector field glyph representation corresponding to chosen zones from the phase diagram: (1) = skyrmionic zone, (2) = perpendicularly magnetized ground-states, (3) = vortex type states, (4), (5) = other complex chiral magnetic structures.

However, for practical applications, it would be interesting to stabilize skyrmions without the need to apply external magnetic fields, especially when these fields are typically large. For this purpose, we show that one can exploit the demagnetizing field in patterned structures, i.e., in patterned circular disks, the demagnetizing field and its symmetry help to stabilize skyrmionic ground states within a complex phase diagram of magnetic parameters. In Figure 9, we illustrate the results of such a micromagnetic simulation performed on circular disks with a 100 nm diameter, corresponding to a 120 nm film like our experimental sputtered films. The saturation magnetization at room temperature is $M_{s}$ = 832 kA/m, as determined from the magnetic analysis of the sputtered films, the other micromagnetic parameters being A_ex_ = 6 pJ/m for the direct exchange constant (in accordance with the literature) and a Gilbert damping parameter α = 0.1. The phase diagram is calculated in the Anisotropy–DMI parameter space, relaxing the system from an initial state with the magnetization saturated along the -Oz axis. One can clearly see that a skyrmionic ground state can be stabilized within a wide parameter window (blue colored zone corresponding to a topological charge Q ≈ −1), including our experimental parameters corresponding to the estimated uniaxial anisotropy K_u_ ≈ 0.35 MJ/m^3^, for which a DMI of about 2 mJ/m^2^ would be required to promote skyrmions in the ground state.

Outside of the skyrmionic window, other interesting chiral structures can be stabilized, as illustrated in Figure 9, e.g., vortices (topological charge *Q* ≈ − 0.5) or even more complex structures with *Q* > 1. An interesting result that can be observed in the calculated phase diagrams is that decreasing the anisotropy leads to a lower DMI, which is necessary for skyrmion stabilization. This is a critical issue, and for the experimental implementation of the phase diagram this aspect must be correlated with the possible origin of the DMI in the ferrimagnetic DyCo_3_ films [48]. Typically, in thinner films the DMI has a surface origin, being induced by proximity effects with adjacent layers: heavy metals with large spin–orbit coupling or hybridization with oxygen from adjacent oxide layers [20]. However, for thick films, different complex mechanisms are involved. Commonly, the bulk DMI has been associated with an asymmetric elemental distribution [19] or the absence of the inversion symmetry center in amorphous materials [21] the consequence of which would be an equivalent inner electric field, that in the referential of the electron would constitute a magnetic field providing an effective spin–orbit interaction mechanism responsible for the DMI.

## 4. Discussion and Conclusions

We successfully fabricated ferrimagnetic bulk DyCo_3_ and thin film DyCo_x_ systems, as demonstrated by the structural (X-ray Diffraction, Figure 4) and magnetic analysis (Figure 5 and Figure 7) in variable field and temperature. The ferrimagnetic configuration of the Dy and Co magnetic moments is confirmed by the good agreement between the measured magnetic moment and the theoretically predicted ones (Table 1).

The theoretical ab initio modelling of the DyCo_3_ system within the DFT formalism allowed us to get deep insight into the atomic contribution to the magnetic properties of a complex ferrimagnetic compound and phenomenologically understand the impact of the local atomic environment on the magnetic moment that explains the relatively broad dispersion of the values reported in the literature for the saturation magnetization per formula unit for DyCo_3_. Moreover, from the theoretical magnetic values expected for Co and Dy compared with the experimentally determined M_s_, we were able to estimate the experimental stoichiometry of the DyCo_x_ thin films, whose deviation from the 1:3 theoretical expectation can be reasonably explained by the different sputtering yields of Dy and Co atoms when sputtering from a stoichiometric target.

The magnetic analysis performed in a temperature range from 4 to 300 K allowed the determination of the temperature variation of the main magnetic parameters such as the saturation magnetization M_s_ and the effective uniaxial anisotropy K_u_. For thin film systems, the magnetic analysis at both microscopic (Magnetic Force Microscopy, Figure 6) and macroscopic (SQUID magnetometry, Figure 7) scales reveals the presence of a perpendicular magnetic anisotropy, as expected from the theoretical calculations for the DyCo_3_ compound with hexagonal crystallographic symmetry (Figure 1, Figure 2 and Figure 3). The PMA-induced maze-like domain wall structure is crucial for magnetic bubble nucleation under a perpendicular magnetic field. With DMI present, these bubbles form skyrmions, and their chirality is determined by the DMI sign. As we could not experimentally measure the DMI—pending Brillouin Light Scattering experiments—we used micromagnetic simulations to predict the skyrmion potential. We considered a typical DMI value for continuous films under a perpendicular field (Figure 8) and calculated theoretical phase diagrams to define/illustrate the conditions for getting the skyrmionic ground state in patterned nanopillars without an external magnetic field (Figure 9). For the micromagnetic simulations, we used magnetic parameters derived from magnetic analysis. Combined with experimental results, the simulations highlight the skyrmionic potential of our samples in both continuous thin films and nano-patterned structures, with direct DMI evaluation planned as a future study.

### 4.1. Intrinsic and Extrinsic Origin of the DMI in DyCo_3_ Samples

The origin of the DMI in DyCo_3_ samples remains a challenging theoretical and experimental issue. The intrinsic mechanisms would be related to the electronic structure in a system with broken inversion symmetry when the spin–orbit interactions are considered. On the other hand, the extrinsic mechanisms would be also consequences of the spin–orbit coupling in a magnetic system with broken inversion symmetry by some structural and chemical distribution issues in realistic samples.

The collinear framework used in our ab initio calculations, provided by the FP-LAPW Wien2k code, does not allow direct evaluation of antisymmetric exchange. An accurate evaluation would require a non-collinear spin framework for spin–spiral calculations in real or reciprocal space, with the DMI determined as the energy difference between two DFT energies corresponding to spiral–spin configurations with opposite rotational senses [49]. The only alternative within our collinear framework, the four-states method [49], is highly complex and difficult to implement for the complex DyCo_3_ supercell. Additionally, the chosen four spin configurations do not correspond to energy minima, and their magnetic symmetry differs. Addressing these issues would require a detailed study of k-point meshing in reciprocal space to prevent missing states that could significantly affect the total energy evaluation. These challenges would impede the convergence of DFT+ Spin–Orbit coupling calculations and hinder accurate DMI evaluation. As a result, ab initio DMI calculations are beyond the scope of this study. However, they offer an interesting theoretical perspective that could reveal potential intrinsic bulk DMI mechanisms in DyCo_3_.

The origin of extrinsic mechanisms related to the structural characteristics of DyCo_3_ is even more complex due to the various possible mechanisms of bulk and interface DMI in RE-TM alloy films combined with the specific non-collinear magnetic structure of DyCo_3_ [17,18,19,20,21,22,23,24]. Moreover, the DyCo_x_ alloys have complex intrinsic non-collinear magnetic ground states in both single-crystal and amorphous films. The rhombohedral structure of DyCo_3_, with two inequivalent Dy sites and three inequivalent Co sites, results in complex non-collinear configurations [25]. At one site, Dy aligns with the easy axis, while at the other, Dy aligns parallel to the c-axis, creating a non-collinear spin arrangement due to the frustrated magnetic anisotropy of rare-earth ions. Such non-collinear spin arrangements have also been observed in amorphous DyCo_3_ by Mössbauer spectroscopy [26], showing a sperimagnetic arrangement for Dy and ferromagnetic order for Co. Experimentally, our as-deposited DyCo_3_ films are expected to exhibit a mixed crystallographic phase, comprising both amorphous and crystalline components. Therefore, within this complex theoretical and experimental context, the precise origin of the DMI in the DyCo_3_ samples (bulk and thin films) remains a challenging issue and investigation prospect.

### 4.2. Prospects

As future directions based on the results presented here, we aim to explore DyCo_x_ films with varying thicknesses, down to a few nanometers. On these systems, we aim to investigate the thickness dependence of the magnetic properties, namely M_s_ and K_u_, and their variation with temperature, and determine the critical DyCo_x_ thickness required for some critical magnetic regimes: weak-stripe and perpendicular stripe stabilization [21]. Additionally, we plan to conduct patterning and micromagnetic analysis (such as MFM and Lorentz microscopy under applied magnetic field) of both continuous film systems and nano-disks to investigate their potential for hosting skyrmionic ground states, with the possibility of using Brillouin light scattering to evaluate the DMI. Finally, these types of studies will be performed on films with variable Co stoichiometry, by co-evaporating from a stoichiometric DyCo_3_ target and additional convergent Co target, using the confocal geometry already available in our sputtering plant.

## Figures and Tables

**Table 1:** Calculated spin, orbital and total magnetic moments for the inequivalent Dy and Co atoms in the supercell.

${Dy}_{1}$		${Dy}_{2}$		${Co}_{1}$		${Co}_{2}$		${Co}_{3}$	
ORB [μ_B_]	SPIN [μ_B_]	ORB [μ_B_]	SPIN [μ_B_]	ORB [μ_B_]	SPIN [μ_B_]	ORB [μ_B_]	SPIN [μ_B_]	ORB [μ_B_]	SPIN [μ_B_]
3.54	6.07	3.56	6.42	−0.12	−1.37	−0.15	−1.59	−0.09	−1.43
9.61		9.98		−1.49		−1.74		−1.52	

## Data Availability

The data presented in this study are available on request from the corresponding author.

## Abbreviations

The following abbreviations are used in this manuscript:

AF	Antiferromagnetic
AFM	Atomic Force Microscopy
DFT	Density Functional Theory
DMI	Dzyaloshinskii–Moriya interaction
FPLAPW	Full Potential Linearized Augmented Plane-Wave
IP	In-plane
LSDA	Local Spin Density Approach
MAE	Magnetic Anisotropy Energy
MFM	Magnetic Force Microscopy
OOP	Out-of-plane
PMA	Perpendicular Magnetic Anisotropy
RE	Rare Earth
SkHE	Skyrmion Hall Effect
SQUID	Superconducting Quantum Interference Device
STT	Spin Transfer Torque
TM	Transition Metal
UHV	Ultra-High-Vacuum
VSM	Vibrating Sample Magnetometer
